# Comparison of Proteome Composition of Serum Enriched in Extracellular Vesicles Isolated from Polycythemia Vera Patients and Healthy Controls

**DOI:** 10.3390/proteomes7020020

**Published:** 2019-05-06

**Authors:** Anna Fel, Aleksandra E. Lewandowska, Petro E. Petrides, Jacek R. Wiśniewski

**Affiliations:** 1 Intercollegiate Faculty of Biotechnology, University of Gdańsk and Medical University of Gdańsk, Abrahama 58, 80-307 Gdańsk, Poland; anna.fel@biotech.ug.edu.pl (A.F.); aleksandra.lewandowska@biotech.ug.edu.pl (A.E.L.); 2 Hematology Oncology Center and Ludwig Maximilians University of Munich Medical School, Zweibrückenstraße 2, 80331 Munich, Germany; petro.petrides@t-online.de; 3 Department of Proteomics and Signal Transduction, Max-Planck-Institute of Biochemistry, Am Klopferspitz 18, 82152 Martinsried, Germany

**Keywords:** extracellular vesicles, polycythemia vera, proteomics, total protein approach, multi-enzyme digestion filter aided sample preparation (MED-FASP)

## Abstract

Extracellular vesicles (EVs), e.g., exosomes and microvesicles, are one of the main networks of intercellular communication. In myeloproliferative neoplasms, such as polycythemia vera (PV), excess of EVs originating from overabundant blood cells can directly contribute to thrombosis through their procoagulant activity. However, the proteomic composition of these vesicles in PV patients has not been investigated before. In this work, we examined the proteomic composition of serum EVs of PV patients in comparison to healthy controls. We processed EV-enriched serum samples using the Multiple Enzyme Filter Aided Sample Preparation approach (MED-FASP), conducted LC-MS/MS measurements on a Q-Exactive HF-X mass spectrometer, and quantitatively analyzed the absolute concentrations of identified proteins by the Total Protein Approach (TPA). Thirty-eight proteins were present at statistically significant different concentrations between PV patients’ study group and healthy controls’ group. The main protein components deregulated in PV were primarily related to excessive amounts of cells, increased platelet activation, elevated immune and inflammatory response, and high concentrations of procoagulant and angiogenic agents. Our study provides the first quantitative analysis of the serum EVs’ proteome in PV patients. This new knowledge may contribute to a better understanding of the secondary systemic effects of PV disease and further development of diagnostic or therapeutic procedures.

## 1. Introduction

The means by which cells within a tissue, an organ, or whole organism communicate play a key role in shaping physiologically relevant cellular responses. Cell-to-cell communication is a fundamental component of both normal physiology and pathophysiology. One of the mechanisms by which cells communicate is the generation and processing of extracellular vesicles [1]. Extracellular vesicles (EVs), including exosomes and microvesicles (MVs), are released by diverse cell types both in healthy and diseased tissues [2,3]. Exosomes promote directed and random cell motility, invasion, and serum-independent growth. These small vesicles (approximately 50–100 nm in diameter), which were present inside large multivesicular endosomes, appear to arise through unfolding and budding from the limiting membrane of late endosomes. The biogenesis of exosomes determines their membrane orientation [4,5]. For instance, exosomes from reticulocytes contain the transferrin receptor, which is absent in mature erythrocytes. The transferrin receptor is a marker of immature erythrocytes, internalized from a plasma membrane, which might be used to follow endocytosis and the recycling of cell surface proteins. This process results in the gain of vesicles that contain the cytosol and which have exposed the extracellular domain of the transferrin receptor on their surface [1,4]. The exosome-mediated information transfer allows for the crosstalk of cells within the hematopoietic system and for interactions between hematopoietic cells and local or distant tissue cells. The molecular and genetic mechanisms that exosomes utilize to shuttle information between cells are currently being examined as well as the potential roles exosomes play as biomarkers of disease or future therapeutic targets [6].

Although the role of EVs in patients with hematologic malignancies has not been studied so extensively as compared to solid cancer studies [7,8,9,10], exosomes have been reported to play a crucial role in every aspect of the communication, development, functional integrity, and progression to the hematologic malignancy [6]. These vesicles can reprogram the bone marrow microenvironment, creating a place for abnormal cells and promoting their expansion. As reported for solid cancer, serum/plasma of patients with hematologic malignancies is enriched in exosomes in comparison with healthy volunteers. Particularly, newly diagnosed patients with acute myeloid leukemia (AML) or chronic lymphocytic leukemia (CLL) have significantly higher serum levels of exosomes (measured by the amount of protein in plasma-isolated exosomes) compared to levels in the serum of healthy controls [11]. Furthermore, increased levels of platelet derived microparticles have been reported in patients with myeloproliferative neoplasms, such as polycythemia vera (PV) [12,13,14,15]. PV is a heterogeneous disease of stem cells with preferential increase in the number of erythrocytes, which through resulting increased viscosity in combination with other pathogenic factors, may lead to thromboembolic complications. This neoplasm is etiologically linked to a somatic mutation in the JAK2 gene (JAK2V617F), which is found in about 90% of patients with PV, and in about 50% of essential thrombocythemia (ET) and primary myelofibrosis (MF) [16]. The World Health Organization (WHO) classified the classic myeloproliferative diseases as neoplastic due to their clonality with additional revisions in 2016 [17]. On the other hand, somatic mutations in the endoplasmic reticulum chaperone gene (CALR) are detectable in a majority of myeloproliferative neoplasms (MPN) patients with non-mutated JAK2 and in a small proportion of MPN somatic mutations in the MPL gene have been also reported [18,19]. There are two mechanisms of a hypercoagulable state associated with PV–firstly, involving the abnormal components of the hematopoietic system (platelets, erythrocytes and leucocytes) which are derived from the clonal proliferation of the hematopoietic progenitor cells and prothrombotic phenotype; secondly, inflammatory response from the host towards cytokines and the inflammatory mediators released by malignant cells [20,21,22]. Although it is known that extracellular vesicles released from cell membranes and circulating in the blood can directly contribute to thrombosis through their procoagulant activity, the potential association between the proteomic composition of EVs and the risk of thrombotic events in PV has not been described before.

**Figure 1:**
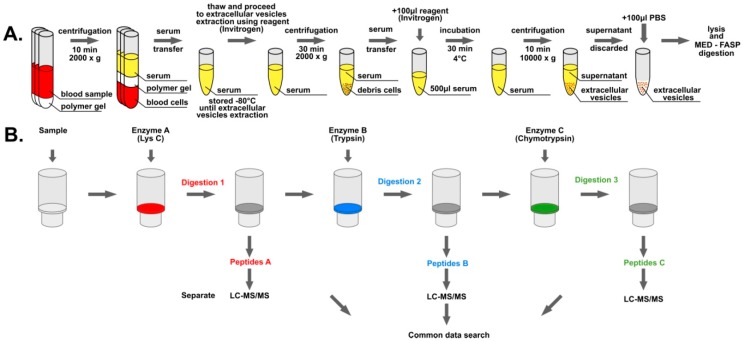
Overview of the analytical workflow: (**A**) Isolation of extracellular vesicles (EVs) from blood samples; (**B**) multi-enzyme digestion filter aided sample preparation (MED-FASP) digestion protocol and liquid chromatography coupled with tandem mass spectrometry (LC-MS/MS) analysis of the samples.

The involvement of EVs in the intercellular communication and molecular programming of different cell types creates a demand for a straight characterization of the proteomes that those vesicles deliver to cells. In this work, we decided to compare the proteomic profiles of EVs purified from the serum of patients suffering from polycythemia vera and healthy volunteers using proteomics methods (see Figure 1), i.e., Multiple Enzyme Filter Aided Sample Preparation (MED-FASP) [23], liquid chromatography coupled with tandem mass spectrometry (LC-MS/MS) measurements, and Total Protein Approach (TPA) [24].

## 2. Materials and Methods 

### 2.1. Blood Collection and Serum Isolation

Blood samples were collected into the 8.5 mL BD Vacutainer™ Venous Blood Collection Tubes: SST™ Serum Separation Tubes. In total, 18 samples were collected: Nine from patients diagnosed with polycythemia vera (diagnosed by WHO 2016 criteria) and nine control samples from healthy donors. All patients were on phlebotomy and aspirin treatment. Each donor gave written consent regarding the use of collected material in translational studies and the publication of obtained results. Examination of patient blood samples had been approved by Ludwig-Maximilians-Universität ethics committee. Leukocyte, platelet, and cholesterol blood concentrations of patients taking part in the study are listed in Supplementary Table S1a. Blood samples were allowed to clot at room temperature for 30 min after collection. The serum was separated from the clot by centrifugation at room temperature for 10 min at 2000 g. Then, the serum was transferred into a new tube, frozen by liquid nitrogen and stored in −80 °C until EV extraction.

### 2.2. Extracellular Vesicle Isolation

EVs were isolated from the blood serum using the Total Exosome Isolation (from serum) reagent (Invitrogen) according to the manufacturer’s procedure (Figure 1a). Samples were thawed at room temperature and centrifuged at 2000 g for 30 min to remove cells and debris. Supernatant containing the clarified serum was transferred to new tubes and placed on ice prior to the procedure. Briefly, 500 µL of serum was mixed with 100 µL of reagent and incubated at 4 °C for 30 min. Afterwards, samples were centrifuged at 10,000 g for 10 min at room temperature. Supernatant was discarded, and the pellet containing EVs was dissolved in 100 µL of PBS buffer. A 1% SDS buffer (100 mM Tris-HCl pH 8, 50 mM DTT, 1% SDS) was used at the ratio of 1:5 (EVs: buffer) for 10 min at 95 °C to lyse obtained EVs and reduce disulfide bonds of released proteins. Lysed EVs were frozen by liquid nitrogen and stored at −20 °C until further processing.

### 2.3. Protein Digestion

Samples containing lysed EVs were subjected to digestion by the MED-FASP (Multi-Enzyme Digestion Filter Aided Sample Preparation) procedure (Figure 1b) [23]. Total protein concentration was measured using the tryptophan fluorescence-based (WF) assay [25] (see Supplementary Table S1b), and 100 µg of total protein was transferred to Microcon 30 kDa filters (Merck). 200 µl of the UA buffer (8 M urea in 100 mM Tris-HCl pH 8.5) was added to samples onto filters and the filters were centrifuged at 10,000 g for 20 min at room temperature. This procedure was repeated two times. Cysteine alkylation was performed by the incubation of samples in darkness at room temperature for 20 min with 100 µL of 55 mM iodoacetamide solution in the UA buffer followed by centrifugation at 10,000 g for 15 min at room temperature. Afterwards, samples were washed three times with 100 µL of the UA buffer and two times with 100 µL of the digestion buffer (50 mM Tris-HCl) by centrifugation in the same conditions. Three consecutive protein digestions with different enzymes were conducted on filters. First, proteins were digested overnight with LysC at 1:50 enzyme to protein ratio in the digestion buffer. Peptides were then eluted by centrifugation at 10,000 g for 15 min at room temperature, followed by two washes with 125 µL and 100 µL of the digestion buffer. Afterwards, filters were placed in new tubes and samples were subjected to digestion by trypsin overnight at 1:100 enzyme to protein ratio in the digestion buffer. Peptides were eluted the same way as it was described for the LysC digestion. After placing filters in new tubes, samples were digested by chymotrypsin for three hours at 1:100 enzyme to protein ratio in 10 mM CaCl2 in 100 mM Tris-HCl pH 7.8. Peptides were eluted by the digestion buffer as described before. All enzymatic digestion reactions were conducted at 37 °C. The peptide concentration was measured by the WF assay. Aliquots containing 10 µg of peptides resulting from each digestion of every sample were separately subjected to STAGE (STop And Go Extraction) TIPS procedure [26] using Empore C18 extraction disks (3M) with elution by 60% acetonitrile/1% acetic acid solution. Samples were concentrated to 5 µL volume in SpeedVac and stored at −20 °C until the MS analysis.

### 2.4. Mass spectra Acquisition and Data Analysis

Liquid chromatography–tandem mass spectrometry measurements of prepared samples were performed using a QExactive HF-X mass spectrometer (ThermoFisher Scientific, Palo Alto, CA, USA) operating in data-dependent acquisition mode, coupled with nanoLC. The raw spectra of all samples were loaded into the MaxQuant software [27] and searched together against the Homo sapiens UniProtKB database (June 2016). Peptides were chromatographed on a 50 cm column with 75 µm inner diameter packed C_18_ material. Peptide separation was carried out at 300 nL/min for 95 min using an acetonitrile gradient of 5–30%. The temperature of the column oven was 55 °C. The mass spectrometer operated in data-dependent mode with survey scans acquired at a resolution of 60,000. Up to the top 15 most abundant isotope patterns with charge ≥ +2 from the survey scan (300–1650 m/z) were selected with an isolation window of 1.4 m/z and fragmented by HCD with normalized collision energies of 25. The maximum ion injection times for the survey scan and the MS/MS scans were 20 and 28 ms, respectively. The ion target value for MS1 and MS2 scan modes was set to 3 × 10^6^ and 10^5^, respectively. The dynamic exclusion time was 30 s. Carbamidomethylation was set as a fixed modification, a maximum of two missed cleavages were allowed, and maximum false discovery rate for peptides and proteins was set at 0.01. Quantification of protein concentrations was conducted using the Total Protein Approach [24] and Max Quant LFQ algorithm [28]. The calculations were performed in Microsoft Excel. Statistical analysis of protein concentrations differing PV patients’ group and healthy controls’ group was conducted in the Perseus software [29] using the PCA analysis and student t-test. The functional gene enrichment analysis and Vesiclepedia database [30] search was performed in the FunRich software [31] using built in FunRich database. The protein interaction analysis was performed in the STRING database [32]. The mass spectrometry proteomics data have been deposited to the ProteomeXchange Consortium [33] via the PRIDE [34] partner repository with the dataset identifier PXD013234.

## 3. Results

### 3.1. General Description of Isolated Extracellular Vesicle Material

**Figure 2:**
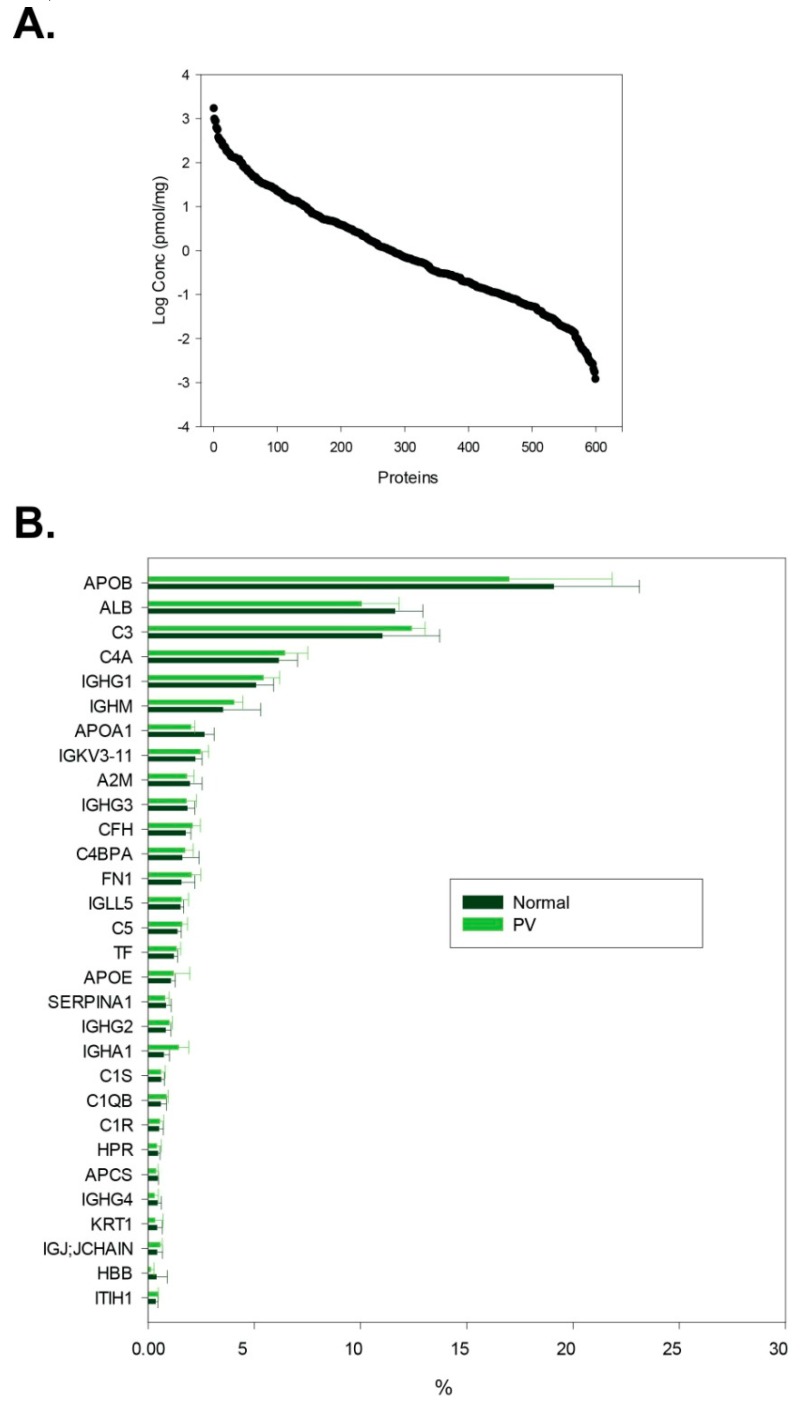
Characterization of isolated material in terms of protein quantity. (**A**) Abundance distribution of all identifications (pmol/mg of isolated material); (**B**) percentage distribution of 30 most abundant proteins in polycythemia vera (PV) patient samples and healthy volunteer samples (median with standard deviation presented).

We identified 624 ± 11 proteins per sample (in total 706); considering only identifications with at least two peptides, we found 513 ± 10 proteins per sample (see Supplementary Table S1c). The concentration range of identified proteins ranges over six orders of magnitude (see Figure 2): from nmol/mg (e.g., serum albumin, immunoglobulin heavy constant gamma 1) to fmol/mg of isolated material (e.g., reelin, myosin-14). An issue complicating the analysis of isolated EVs is the co-precipitation of lipoprotein particles and proteins abundant in the serum, such as serum albumin and immunoglobulins [35]. The serum albumin content has been reduced to about 12% of the isolated material, approximately 5-fold decrease in reference to the typical healthy human serum sample [36], while apolipoproteins were enriched with their content rising to as much as 20% of all present proteins (see Figure 2).

We conducted the comparative analysis of identified proteins using the Vesiclepedia database [30] to determine the EV proteins distribution in our experiments. 466 of 537 recognized by the database proteins were previously reported in the full Vesiclepedia database (by protein evidence), 451 were reported in serum/plasma vesicles, and 407 were reported in serum/plasma exosomes. Most of the unreported proteins belong to the immunoglobulin family. We were able to find 36 out of 100 most often reported EV proteins as listed by the Vesiclepedia resource, 36 out of 100 proteins most often reported in serum/plasma vesicles, and 34 in serum/plasma exosomes. We compared our results with the EV protein markers list presented by C. Théry and K. W. Witwer et al. [35]. We found several EV specific markers and many of the proteins associated with EVs (see Table 1). The prevalence of platelet- and monocyte-specific EV protein markers suggests the presence of vesicles related to those cell types. The absence of some of the most popular exosome markers (e.g., tetraspanins) may indicate contamination of isolated exosomes with other types of EVs as well as different cell structures, therefore we refer to the isolated material in general as extracellular vesicles.

### 3.2. Functional Categorization of Identified Proteins

**Figure 3:**
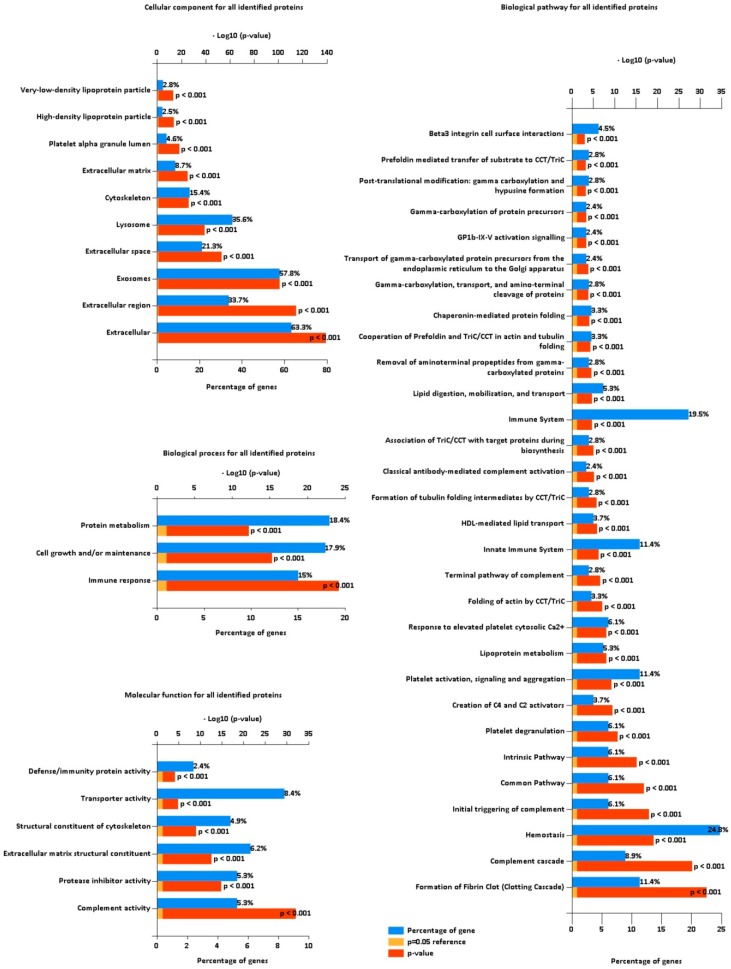
Functional gene enrichment analysis of all identified proteins from the FunRich software for cellular component, biological process, molecular function, and biological pathway. Blue bars represent the percentage of protein genes assigned to the indicated term, yellow bars show the reference p value (0.05), and red bars show the calculated p value of enrichment for the indicated term.

In order to further characterize the isolated proteome, we performed the gene enrichment analysis on the set of all identified proteins (see Figure 3). As expected, cellular components for the majority of identifications were determined to be extracellular (63.3%) and exosomes (57.8%). A substantial part of proteins were localized in lysosomes (35.6%) or cytoskeleton (15.4%), suggesting co-precipitation of those cellular components with EVs or supposed transportation of protein cargo related to those structures. About 3% of all identifications were localized in lipoprotein particles. Even though those proteins represent a small fraction of all identifications, apolipoproteins are among the highest abundant proteins in the isolated material (as it was discussed in Results Section 3.1.). Identified proteins were confidently assigned (*p*-value<0.001) to three biological processes: Protein metabolism (18.4%), cell growth and/or maintenance (17.9%), and immune response (15%). Prevalent molecular functions of identified proteins were assigned as transporter activity (8.4%), extracellular matrix structural constituent (6.2%), complement activity (5.3%), and protease inhibitor activity (5.3%). Designated biological pathways are closely related to blood components: Hemostasis (24.8%), immune system (19.5%), clotting cascade (11.4%), platelet activation, signaling and aggregation (11.4%), and complement cascade (8.9%).

### 3.3. Differences in PV Patients’ Exosomal Proteomes

**Figure 4:**
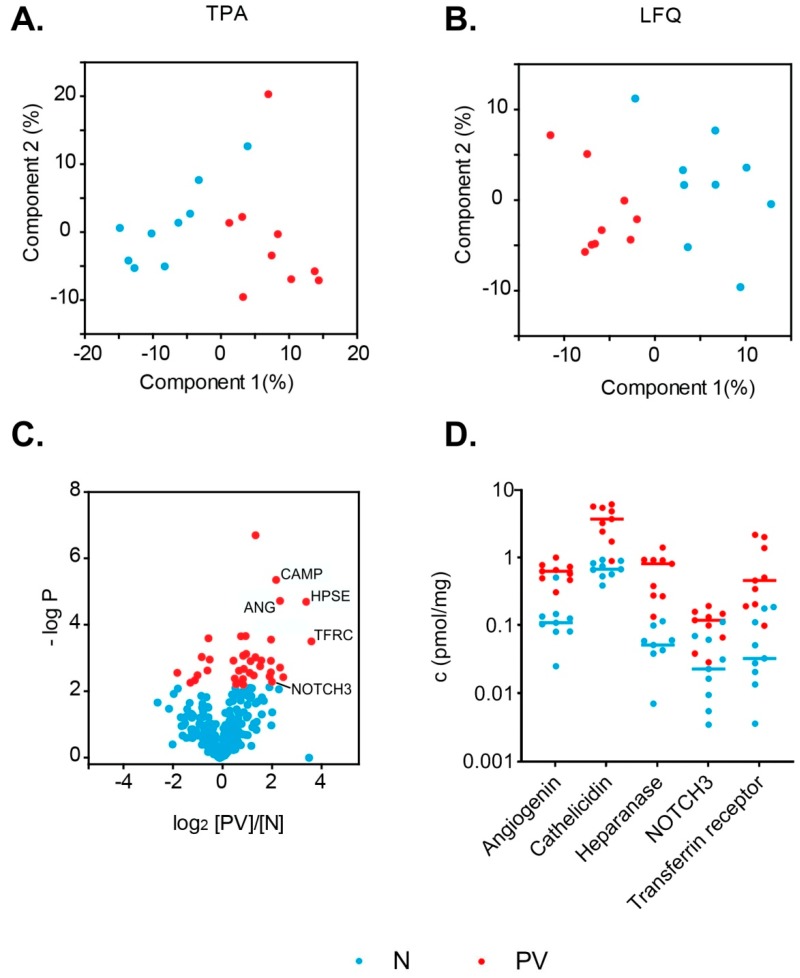
Results of statistical t-test analysis conducted for two groups of samples: PV patient samples and healthy volunteer samples: PCA plots for study samples created from the results of two computational quantitative approaches, (**A**) the TPA and (**B**) Max Quant LFQ quantification; (**C**) the volcano plot showing the experimental outlier proteins (38 proteins); (**D**) concentration plot of six outlier proteins differentiating across the samples (angiogenin–ANG, cathelicidin antimicrobial peptide–CAMP, heparanase—HPSE, neurogenic locus notch homolog protein 3–NOTCH3, and transferrin receptor protein 1—TFRC).

We compared two methods of label-free protein quantification: Total Protein Approach [24] and MaxQuant LFQ [28]. We quantified only the proteins with at least three razor or unique peptides, and eight out of nine valid peak intensity values in one of the analyzed study groups (PV patients or healthy controls). Using this approach, 398 distinct protein groups were quantified (see Supplementary Table S1d). We performed the principal component analysis of studied samples using quantitative values obtained using both methods (absolute concentration in pmol/mg from TPA, see Figure 4a, and LFQ intensity from MQ LFQ, see Figure 4b). We compared protein concentration differences observed with TPA and MQ LFQ approaches (Supplementary Table S1e). We found that 24 significantly changed protein titers were common for both methods. The calculated titer ratios correlated with a Pearson correlation coefficient r = 0.80. Both of those methodologies reveal differences of similar magnitude between study groups. For a further in-depth quantitative analysis we used absolute concentration values obtained from TPA.

Approximately 10% of quantified proteins were present at different concentrations (at least 1.5-fold) between study groups from a student t-test analysis at 5% FDR cutoff: 30 proteins were more abundant in PV patient samples and eight proteins were less abundant (see Table 2). The presence of higher abundance of erythrocyte (CD71), platelet (CD42d), and monocyte (CD62L) membrane markers may suggest an elevated extracellular vesicle count of those cell types in polycythemia vera. This observation is further supported by elevated leukocyte and platelet counts in PV patients (see Supplementary Table S1a). Twenty proteins were present at more than 2-fold higher concentrations in PV samples, and 11 of them higher than 4-fold. Only three proteins had at least 2-fold lower concentrations in PV samples (see Figure 4c). Five proteins are marked on the volcano plot and their concentration differences are presented in Figure 4d: Angiogenin (ANG), cathelicidin antimicrobial peptide–CAMP, heparanase–HPSE, neurogenic locus notch homolog protein 3–NOTCH3, and transferrin receptor protein 1–TFRC. Overabundance of those proteins suggests deregulation of several different biological processes in PV, either by excessive numbers of distinct cell types or overstimulation of certain pathways. The protein that displays the highest difference in concentrations (13-fold) among study groups is the transferrin receptor protein 1 (TRFC).

**Figure 5:**
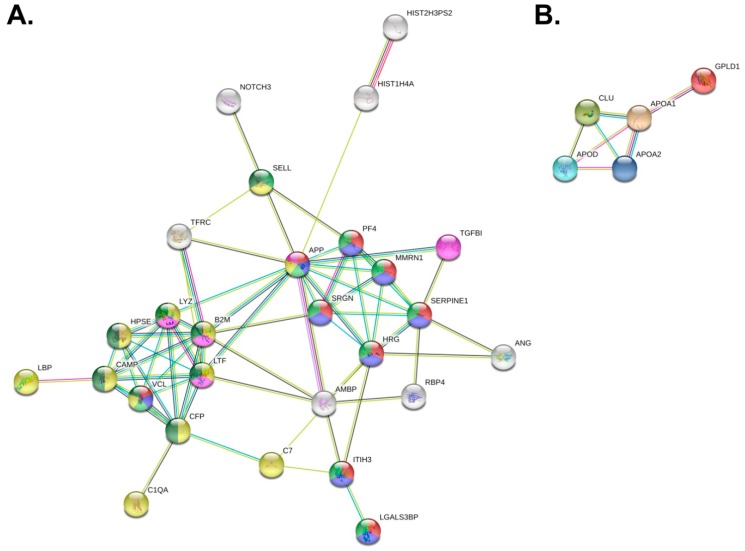
Interaction networks of proteins present at statistically significant different concentrations in PV samples inferred from the t-test at 5% FDR built using the STRING database. Proteins absent from the networks are not shown. (**A**) Interaction network of proteins present at higher concentrations in PV samples. Colors of nodes designate involvement in assigned Reactome pathways: Red–platelet degranulation, blue–platelet activation, signaling and aggregation, light green–hemostasis, yellow–innate immune system, purple–amyloid fiber formation, and dark green–neutrophil degranulation; (**B**) interaction network of proteins present at lower concentrations in PV samples.

We performed an interaction analyses of proteins significantly differing between patient and control groups using the STRING database [32] to seek potential relations between them. Both more abundant proteins and less abundant proteins constituted networks of significantly more interactions than expected from random sets of proteins (PPI enrichment values of lower than 1.0 × 10^−16^ and 2.78 × 10^−7^, respectively; see Figure 5). Only three out of 30 proteins enriched in PV samples are absent from the constructed interaction network. Key Reactome database [37] pathways are presented using different node colors on the created network (Figure 5). The network can be visibly divided into two smaller tightly connected systems. The first one is related to the immune response and partly to related neutrophil degranulation (see yellow and dark green nodes in Figure 5a). Upregulation of those pathways may be explained by leukocytosis, JAK2 activation, or excess of granulocytes reported in PV cases [22]. Many proteins or genes from the described system have been previously found to be more abundant or upregulated in PV. Two of those proteins were mentioned previously as the volcano plot outliers (see Figure 4c,d): CAMP and HPSE. The other visible net of interactions is associated with platelet degranulation, activation, signaling and aggregation, and hemostasis (see red and blue nodes in Figure 5a). The presence of such interaction pathways is expected in PV, which is characterized by the persistent platelet activation and excessive cell adhesiveness [22]. The network for proteins present at lower concentrations in PV samples constituted of five out of eight proteins (see Figure 5b). The main characteristics of these proteins are constituents of lipoprotein particles, lipoprotein particle receptor binding, and reverse cholesterol transport (APOAI, APOAII, CLU) and cholesterol binding (APOAI, APOAII, APOD).

## 4. Discussion

Polycythemia vera, one of the Philadelphia chromosome negative myeloproliferative neoplasms, is characterized by increased bone marrow hematopoiesis, resulting in the overproduction of erythrocytes, and frequently also of platelets and leukocytes. A typical complication of this disorder is thrombosis, which is a result of the hypercoagulable state associated with excessive amounts of blood cells and inflammatory response [20,21,22]. PV is associated with persistent enhanced platelet activation. When activated, platelets release extracellular vesicles: Mainly procoagulant microvesicles and exosomes [12]. In our work, we isolated and studied EV-enriched sera of PV patients in comparison to healthy volunteers. EVs, such as exosomes, are one of the main channels of inter-cell communication. Although it is known that the previously reported excess of extracellular vesicles released from cell membranes of activated or apoptotic blood cells directly contribute to thrombosis through their procoagulant activity [12,13,14], the exact proteomic composition of these vesicles has not been investigated previously. The exact mechanisms of polycythemia vera occurrence, course, and possible leukemic transformation are not known. By studying proteomes of extracellular vesicles released in PV, we managed to get a closer look into the secondary systemic effects of the disease. We identified the main protein components of EVs deregulated in PV, which were related with excessive amounts of cells (TFRC, SELL, GP5), increased platelet activation (SERPINE1, MMRN1), elevated immune and inflammatory response (HPSE, CAMP, LYZ, SELL, LTF), high concentrations of procoagulant and angiogenic agents (ANG, HPSE), as well as oncogenic proteins (NOTCH3). 

We have found higher concentrations of erythrocyte, platelet, and leukocyte surface membrane markers, supporting previous discoveries of the abundance of those blood cells in PV [22]. This finding was also verified by PV patients’ leukocyte and platelet elevated counts (Supplementary Table S1a), together suggesting the prevalence of serum EVs related to those cell types. Transferrin receptor protein 1 (TFRC, CD71), a reticulocyte marker, was found to be as much as 13-fold more abundant in samples of PV patients, illustrating massive erythrocyte overproduction in the disease. During reticulocyte maturation, TFRC is removed to exosomes [1]. One of the megakaryocyte membrane markers, translocated to platelets upon their release, is platelet glycoprotein V (GP5, CD42d); here, found to be more than two times more abundant in PV samples. About one third of proteins identified at higher concentrations in PV were associated with platelet degranulation, activation, signaling, and aggregation (APP, HRG, ITIH3, LGALS3BP, MMRN1, PF4, SERPINE1, SRGN, VCL), and most of them were tightly connected in the interaction network (Figure 5a). Plasminogen activator inhibitor 1 (SERPINE1) was almost 6-fold more abundant in PV samples, and its mRNA was previously reported to be upregulated in PV and associated with thrombotic risk and inflammatory pathways [38]. Multimerin-1 (MMRN1) is also tightly associated with platelet release as a component of the thrombopoietin receptor. Surprisingly, one of the higher abundant proteins, serglycin (SRGN) was previously reported as a marker of AML, not upregulated in PV, and therefore allowing distinction between those two blood disorders [39]. However, there are reports of interaction between serglycin and lysozyme [40], which indicate that their concentrations could be correlated in this instance. Even more proteins present at higher concentrations in PV take part in immune response (APP, B2M, C1QA, C7, CAMP, CFP, HPSE, LBP, LTF, LYZ, SELL, VCL). L-selectin (SELL, CD62L), closely related to the granulocyte count, was present at more than four times higher concentrations in PV samples. Lysozyme and lactotransferrin concentrations were previously reported to be elevated in the serum of PV patients [40,41]. Moreover, lysozyme may be stored in megakaryocytes, which seem to be present in excessive amounts in PV as their marker CD42d is increased, as well as overall platelet count. Cathelicidin (CAMP) is associated with granules released upon platelet activation, which closely correlates with inflammatory response activation in PV patients. Lipopolysaccharide-binding protein (LBP) interacts with CAMP and is similarly involved in the immune response. Beta-2-microglobulin is a component of MHC I class molecules. Its level may be raised in myeloproliferative neoplasms [42]. Another key protein, heparanase (HPSE) was previously reported to be present at higher concentrations in PV bone marrow biopsies [43], is associated with myelofibrosis and displays procoagulant and angiogenic activity. HPSE concentration in PV patient samples was elevated more than 11-fold. Increased HPSE activity is associated with the formation of blood borne tumor metastases. Other upregulated angiogenic proteins are, e.g., ANG, HRG, or ITIH3. Angiogenin (ANG) promotes angiogenesis and tumor growth and was found to be overexpressed in leukemia [44]. Its concentration was elevated more than 5-fold in PV patients. One more significant protein, absent from previously described pathways is the neurogenic locus notch homolog protein 3 (NOTCH3), found at more than 4-fold increased concentrations in PV samples. NOTCH3 was previously found to be overexpressed in several types of cancers [45]. Its excessive concentration in the serum is correlated with hypertrophy of smooth muscle cells in blood vessels as a major cause of the CADASIL syndrome (cerebral autosomal dominant arteriopathy with subcortical infarcts and leukoencephalopathy) [46].

In order to easily isolate extracellular vesicles in high concentrations, we used a precipitation kit, employing the methodology applied and validated before in a number of EV studies [47,48]. In order to remain impartial to experimental results, we focused on the in-depth wide proteomic analysis of the material instead of antibody-based targeted tests. A limitation of our study is the lack of detailed characterization of isolated EVs, which could further improve the understanding of their protein cargo importance in the disease. Using our approach, we have identified a number of EV-related proteins, accompanied by high concentrations of reportedly co-precipitated structures’ components, e.g., serum albumin, apolipoproteins. In fact, most of the proteins present at lower concentrations in PV were associated with lipoprotein particles (APOAI, APOAII, APOD, CLU). Obviously, the presence of those proteins in the isolated EV material is unwelcome, and the problem with lipoprotein contamination of exosomes has been reported before [35]. Nevertheless, the fact that those proteins were less abundant in PV patients may be associated with reports of hypocholesterolemia occurring in PV, associated with a drop in apolipoprotein A-I and B concentrations and possible sequestration of cholesterol to overabundant erythrocytes [49]. Similarly, to apolipoproteins, the presence of histones in the isolated material was not fully anticipated; yet, their upregulation in PV samples could be linked to the upregulation of apoptotic pathways in the disease. However, as the process of protein co-precipitation with EVs is not fully understood, the role of reportedly contaminating proteins in the disease can only be a subject of assumptions.

The pathophysiology of polycythemia vera is still to be delineated: As we have shown in this work, extracellular vesicles, the communication vectors between cells, carry an abundance of versatile protein cargo. Studied here EVs proteome is associated with crucial processes, which undergo in blood cells and their surroundings, such as cell proliferation and differentiation, immune and inflammatory response, or tumor creation and its environment regulation by angiogenesis.

## Figures and Tables

**Table 1:** List of EV protein markers and proteins associated with EV experiments identified in this study along with their concentrations in PV patients and healthy control samples.

Uniprot Accession	Gene Name	Protein Name	Median Concentration [pmol/mg]	
			Controls (N)	Patients (PV)
**Transmembrane or GPI-anchored proteins associated to plasma membrane and/or endosomes ^1^**				
*a)* *Non-tissue specific*				
P08514	ITGA2B	Integrin alpha-IIb (CD41)	1.03 × 10^−1^	7.57 × 10^−2^
P05106	ITGB3	Integrin beta-3 (CD61)	5.46 × 10^−2^	8.54 × 10^−2^
*b)* *Tissue-specific*				
P05067	APP	Amyloid-beta precursor protein	5.29 × 10^−1^	1.35
P08571	CD14	Monocyte differentiation antigen CD14	3.14 × 10^−1^	4.96 × 10^−1^
P14770	GP9	Platelet glycoprotein IX (CD42a)	8.09 × 10^−2^	5.77 × 10^−2^
P08514	ITGA2B	Integrin alpha-IIb (CD41)	1.03 × 10^−1^	7.57 × 10^−2^
**Cytosolic proteins recovered in EVs ^1^**				
*a)* *With lipid or membrane protein-binding ability*				
P04083	ANXA1	Annexin A1	Not quantifiable	Not quantifiable
P61586	RHOA	Transforming protein RhoA	Not quantifiable	Not quantifiable
*b)* *Promiscous incorporation in EVs*				
P60709	ACTB	Actin, cytoplasmic 1	3.38	3.84
P63267	ACTG2	Actin, gamma-enteric smooth muscle	2.68 × 10^−1^	5.59 × 10^−1^
P04406	GAPDH	Glyceraldehyde-3-phosphate dehydrogenase	1.84 × 10^−1^	2.83 × 10^−1^
P68366	TUBA4A	Tubulin alpha-4A chain	9.78 × 10^−2^	5.54 × 10^−2^
P07437	TUBB	Tubulin beta chain	1.93 × 10^−2^	Not quantifiable
Q9H4B7	TUBB1	Tubulin beta-1 chain	2.75 × 10^−2^	3.77 × 10^−3^
**Major components of non-EV co-isolated structures (abundant in plasma, serum) ^1^**				
P02768	ALB	Serum albumin	1.67 × 10^3^	1.45 × 10^3^
P02647	APOA1	Apolipoprotein A-I	8.57 × 10^2^	6.53 × 10^2^
P02652	APOA2	Apolipoprotein A-II	1.77 × 10^2^	1.26 × 10^2^
P04114	APOB	Apolipoprotein B-100	3.70 × 10^2^	3.29 × 10^2^
**Transmembrane, lipid-bound and soluble proteins associated to other intracellular compartments than PM/endosomes ^1^**				
*a)* *Nucleus*				
P33778	HIST1H2BB	Histone H2B type 1-B	8.83 × 10^−2^	2.73 × 10^−1^
P62805	HIST1H4A	Histone H4	2.98 × 10^−1^	2.83
*b)* *Secretory pathway (endoplasmic reticulum, Golgi apparatus)*				
P11021	HSPA5	Endoplasmic reticulum chaperone BiP	4.86 × 10^−1^	7.16 × 10^−1^
*c)* *Others (autophagosomes, cytoskeleton, ...)*				
P12814	ACTN1	Alpha-actinin-1	1.89 × 10^−1^	2.97 × 10^−1^
**Secreted proteins recovered with EVs ^1^**				
*a)* *Cytokines and growth factors*				
Q9GZP0	PDGFD	Platelet-derived growth factor D	Not quantifiable	8.34 × 10^−3^
P01137	TGFB1	Transforming growth factor beta-1 proprotein	3.47 × 10^−1^	1.01
*b)* *Adhesion and extracellular matrix proteins*				
P02765	AHSG	Alpha-2-HS-glycoprotein	1.39 × 10^1^	1.07 × 10^1^
O43866	CD5L	CD5 antigen-like	7.59 × 10^1^	6.59 × 10^1^
Q99715	COL12A1	Collagen alpha-1(XII) chain	9.68 × 10^−3^	1.03 × 10^−2^
P39060	COL18A1	Collagen alpha-1(XVIII) chain	1.33 × 10^−1^	1.04 × 10^−1^
P12109	COL6A1	Collagen alpha-1(VI) chain	4.09 × 10^−2^	2.25 × 10^−2^
P12111	COL6A3	Collagen alpha-3(VI) chain	8.48 × 10^−2^	1.37 × 10^−1^
P02751	FN1	Fibronectin	5.91 × 10^1^	7.78 × 10^1^
Q08380	LGALS3BP	Galectin-3-binding protein	5.23	1.07 × 10^1^

^1^ Protein classification presented here directly refers to EV protein markers list presented by C. Théry and K. W. Witwer et al. [35].

**Table 2:** Significantly changed proteins from the t-test analysis at 5% FDR along with their concentrations and fractions of total protein in patient and control samples.

Protein	Gene	*p* Value	[PV]/[N] ^1^	Peptides	Conc. (pmol/mg)		Fraction of Total Protein%	
					N	PV	N	PV
Transferrin receptor protein 1	TFRC	3.30 × 10^−4^	13	19	0.03	0.46	2.80 × 10^−4^	3.91 × 10^−3^
Heparanase	HPSE	2.10 × 10^−5^	11	11	0.05	0.82	3.18 × 10^−4^	5.00 × 10^−3^
Plasminogen activator inhibitor 1	SERPINE1	3.90 × 10^−3^	5.9	6	0.05	0.37	2.39 × 10^−4^	1.66 × 10^−3^
Angiogenin	ANG	2.00 × 10^−5^	5.4	3	0.11	0.63	1.83 × 10^−4^	1.04 × 10^−3^
Histone H4	HIST1H4A	2.00 × 10^−3^	5.4	5	0.3	2.83	3.38 × 10^−4^	3.21 × 10^−3^
Cathelicidin antimicrobial peptide	CAMP	4.60 × 10^−6^	4.9	5	0.68	3.73	1.31 × 10^−3^	7.20 × 10^−3^
Neurogenic locus notch homolog protein 3	NOTCH3	5.30 × 10^−3^	4.3	8	0.02	0.12	5.62 × 10^−4^	2.93 × 10^−3^
Lysozyme C	LYZ	2.90 × 10^−4^	4.2	8	0.82	2.44	1.35 × 10^−3^	4.03 × 10^−3^
Histone H3	HIST2H3PS2	1.20 × 10^−3^	4.2	4	0.28	1.27	4.38 × 10^−4^	1.96 × 10^−3^
L-selectin	SELL	2.80 × 10^−3^	4.2	4	0.05	0.21	2.31 × 10^−4^	8.66 × 10^−4^
Lactotransferrin	LTF	3.70 × 10^−3^	4	23	0.27	1.09	2.10 × 10^−3^	8.54 × 10^−3^
Vinculin	VCL	1.20 × 10^−3^	3.2	13	0.08	0.2	9.44 × 10^−4^	2.43 × 10^−3^
Multimerin-1	MMRN1	1.80 × 10^−3^	3.1	32	0.39	1	5.35 × 10^−3^	1.38 × 10^−2^
Beta-2-microglobulin	B2M	2.10 × 10^−7^	2.7	6	2.52	6.83	3.46 × 10^−3^	9.37 × 10^−3^
Nidogen-2	NID2	9.80 × 10^−3^	2.7	6	0.02	0.04	2.57 × 10^−4^	6.52 × 10^−4^
Amyloid beta A4 protein	APP	3.40 × 10^−4^	2.6	12	0.53	1.35	4.60 × 10^−3^	1.18 × 10^−2^
Serglycin	SRGN	1.30 × 10^−3^	2.4	5	1.51	3.25	2.66 × 10^−3^	5.73 × 10^−3^
Platelet glycoprotein V	GP5	2.90 × 10^−3^	2.3	11	0.18	0.57	1.11 × 10^−3^	3.49 × 10^−3^
Retinol-binding protein 4	RBP4	2.30 × 10^−4^	2.1	13	4.65	9.59	1.07 × 10^−2^	2.21 × 10^−2^
Lipopolysaccharide-binding protein	LBP	7.80 × 10^−4^	2.1	12	1.44	3.32	7.69 × 10^−3^	1.77 × 10^−2^
TGFβ-induced protein ig-h3	TGFBI	2.20 × 10^−3^	2	12	0.19	0.41	1.41 × 10^−3^	3.07 × 10^−3^
Properdin	CFP	8.80 × 10^−4^	1.9	19	6.74	12.83	3.45 × 10^−2^	6.58 × 10^−2^
Galectin-3-binding protein	LGALS3BP	4.10 × 10^−3^	1.9	27	5.23	10.74	3.42 × 10^−2^	7.02 × 10^−2^
Inter-alpha-trypsin inhibitor heavy chain H3	ITIH3	6.10 × 10^−3^	1.9	29	1.53	3.15	1.53 × 10^−2^	3.15 × 10^−2^
Alpha-1-microglobulin	AMBP	2.2 × 10^−4^	1.8	17	7.75	14.69	3.02 × 10^−2^	5.73 × 10^−2^
Platelet factor 4	PF4	5.60 × 10^−3^	1.8	7	124.41	288.88	1.35 × 10^−1^	3.13 × 10^−1^
Complement component C7	C7	2.50 × 10^−3^	1.7	47	11.11	21.7	1.04 × 10^−1^	2.03 × 10^−1^
Complement C1q subcomponent subunit A	C1QA	1.20 × 10^−3^	1.5	18	72.33	114.86	1.88 × 10^−1^	2.99 × 10^−1^
Histidine-rich glycoprotein	HRG	4.00 × 10^−3^	1.5	22	18.28	31.2	1.09 × 10^−1^	1.86 × 10^−1^
N-acetylmuramoyl-L-alanine amidase	PGLYRP2	5.90 × 10^−3^	1.5	17	2.24	3.37	1.39 × 10^−2^	2.10 × 10^−2^
Clusterin	CLU	1.10 × 10^−3^	0.75	19	31.13	23.19	1.63 × 10^−1^	1.22 × 10^−1^
Apolipoprotein A-I	APOA1	2.70 × 10^−3^	0.73	41	857.47	652.78	2.64	2.01
Apolipoprotein A-II	APOA2	2.40 × 10^−3^	0.71	11	177.37	126.08	1.98 × 10^−1^	1.41 × 10^−1^
Apolipoprotein D	APOD	9.60 × 10^−4^	0.6	13	61.85	37.16	1.49 × 10^−1^	8.98 × 10^−2^
Fibulin-1	FBLN1	3.40 × 10^−3^	0.54	20	3.01	1.63	2.24 × 10^−2^	1.21 × 10^−2^
Phosphatidylinositol-glycan-specific phospholipase D	GPLD1	4.60 × 10^−3^	0.5	9	0.19	0.11	1.74 × 10^−3^	1.04 × 10^−3^
Peroxiredoxin-6	PRDX6	5.50 × 10^−3^	0.44	4	4.45	2.93	1.11 × 10^−2^	7.35 × 10^−3^
Salivary acidic proline-rich phosphoprotein 1/2	PRH1	2.90 × 10^−3^	0.3	3	0.19	0.06	3.23 × 10^−4^	1.01 × 10^−4^

^1^ Fold change (Patients [PV]/Controls [N].

## Supplementary Materials

The following are available online at https://www.mdpi.com/2227-7382/7/2/20/s1, Table S1: Mass spectrometry data: (a) Blood data of PV patients, (b) concentration of isolated EV samples and the total amount of protein content isolated, (c) list of all identified proteins along with their concentrations in samples, (d) results of the statistical t-test analysis, (e) comparison of TPA and MQ LFQ methodologies on proteins significantly differing in concentration between analyzed groups.
